# Diversity of *Mycobacterium tuberculosis *genotypes circulating in Ndola, Zambia

**DOI:** 10.1186/1471-2334-10-177

**Published:** 2010-06-17

**Authors:** Chanda Mulenga, Isdore C Shamputa, David Mwakazanga, Nathan Kapata, Françoise Portaels, Leen Rigouts

**Affiliations:** 1 Tropical Diseases Research Center, P.O. Box 71769, Ndola, Zambia; 2 Institute of Tropical Medicine, 2000 Antwerpen, Belgium; 3 Ministry of Health, National Tuberculosis and Leprosy Program, Lusaka, Zambia; 4 Department of Pharmaceutical, Veterinary and Biomedical Sciences, University of Antwerp, 2000 Antwerpen, Belgium; 5 Tuberculosis Research Section, National Institutes of Health, LCID/NIAID, Bethesda, MD 20892, USA

## Abstract

**Background:**

Tuberculosis (TB) is one of the major public health problems in Zambia. However, information about lineages of *M. tuberculosis *complex (MTBC) isolates useful for epidemiology investigations is unknown. In this study, we investigated the diversity of MTBC isolates from Ndola, a typical Zambian urbanized city with a documented high HIV prevalence.

**Methods:**

This was part of a prospective cohort study in subjects with sputum smear-positive pulmonary TB. Spoligotyping was used to genotype the MTBC isolates and establish the circulating lineages. The 15-locus Mycobacterial Interspersed Repetitive Units - Variable Number Tandem Repeats (MIRU-VNTR) typing was used to study recent transmission.

**Results:**

A total of 98 different spoligotypes were identified among 273 MTBC isolates. The majority (64.8%) of the isolates belonged to 9 known families, while 96 (35.2%) of the isolates were orphans. While LAM (41.8%) was the largest spoligotype family observed, most of the isolates (87.7%) belonging to the SAF1 family, with a significant portion coming from the T (13.6%), and X (5.9%) families. A few isolates (3.6%) belonged to the CAS, EAI, H, S, X1-LAM9 or U families. MIRU-VNTR typing was highly discriminatory (h = 0.988) among the 156 isolates tested in our sample, and increased the discrimination among 82 SAF1 isolates from 6 to 46 distinct patterns. In addition, 3.2% (5/156) of cases with available MIRU-VNTR results harbored more than one MTBC strain.

**Conclusions:**

Our findings show a limited diversity of MTBC in Ndola with a high clustering rate (37.7%), which indicates that recent transmission plays an appreciable role in the dynamics of TB disease in this setting. This conclusion emphasizes the importance of early diagnosis and timely treatment. The results also confirm that MIRU-VNTR typing is suitable for studying the molecular epidemiology of TB in Ndola.

## Background

Zambia is ranked among the world's top 10 high TB incidence countries with an incidence rate of 280 smear-positive tuberculosis (TB) cases per 100,000 inhabitants [1]. The World Health Organization (WHO) estimates the prevalence of all forms of TB in Zambia at 707/100,000 [1]. According to the National Tuberculosis Leprosy Program (NTLP), in 2004, the Copperbelt Province was responsible for nearly a third (27.6%) of the nation's notified TB cases. It was also one of the provinces with the highest Human Immunodeficiency Virus (HIV) prevalence (17%) in Zambia [2]. While efforts have been made to identify drivers of the HIV pandemic in Zambia, similar efforts for the TB epidemic lag far behind. A number of surveillance activities, both biological and socio indicator - studies, which are useful in identifying the risk factors that play a role in driving the HIV epidemic in Zambia, have been implemented. However, efforts in TB have mainly relied on generic recommendations to prevent TB. The epidemiology of TB in Zambia still remains largely unknown, and consequently, epidemiological data cannot help focus TB control strategies. In high TB incidence settings, determination of distinct transmission patterns is often indefinable, but may be greatly enhanced by the use of both molecular and conventional epidemiological tools.

Use of molecular markers for strain-specific differentiation of *Mycobacterium tuberculosis*-complex (MTBC) isolates in epidemiological studies became available in the last decades. Some of the more popular MTBC typing methods being used include IS*6110*-based restriction fragment length polymorphism (RFLP) [3] and PCR-based methods like spoligotyping [4], mycobacterial interspersed repetitive units - variable number of tandem repeats (MIRU-VNTR) [5-8], single-nucleotide polymorphisms [9,10] and large-sequence polymorphism analysis [11-14]. Furthermore, apart from differentiating MTBC strains, MIRU-VNTR typing can easily identify mixed infections in patient isolates. Even so, the choice of typing methods used in studies should be considered carefully to provide meaningful analysis because their ability to discriminate MTBC in different settings varies widely. In addition, knowledge on the (over-) representation of specific genotype families in a community can be important, especially if these families have been implicated in disease complications like drug resistance, severe disease, or increased transmissibility.

In this study, we set out to investigate the circulating MTBC isolates, and use spoligotyping and 15-locus MIRU-VNTR typing to distinguish MTBC isolates from Ndola, an urban city on the Copperbelt Province of Zambia.

## Methods

### Study setting and population

This was part of a prospective cohort study in subjects with sputum smear-positive pulmonary TB, conducted in the Ndola urban district on the Copperbelt Province of Zambia. The Ndola District Health Management Team (DHMT) is responsible for health care service delivery in the district and has a catchment population of about 374,750 persons [15]. The district has 21 health centres, 3 military clinics, and 2 tertiary care hospitals. Most of these health centres are able to deliver TB treatment and care and are referred to as treatment centres, but only 6 are able to provide laboratory services for smear microscopy and are referred to as diagnostic centres.

For this study, sputum samples were collected from all consecutive sputum smear-positive pulmonary TB subjects at 4 of the 6 existing TB diagnostic centres between January and July 2006 as per routine. Both previously-untreated (new) and previously-treated (retreatment) cases were enrolled. The two clinics not included in the study were left out mainly because of their comparatively low population catchment areas at the time and because of logistical problems. All TB patients were treated according to the national TB guidelines [16] in line with the WHO treatment guidelines [17]. Smear-negative subjects were excluded because of logistical limitations such as budgetary and manpower constraints.

### Laboratory methods

After routine microscopy, sputum smear-positive samples were stored in cetylpyridinium chloride (CPC) transport medium and kept at ambient temperature until they were taken to the Tropical Diseases Research Centre (TDRC) on a weekly basis. The samples were later transported from TDRC to the Chest Diseases Laboratory (CDL), a reference laboratory in Lusaka, for culture on Löwenstein-Jensen (LJ) medium following decontamination using the Petroff method [18]. Culture tubes were incubated at 37°C and were read weekly for growth for at least eight weeks. Successfully grown cultures were transported back to TDRC for storage and onward transportation of isolates to the Institute of Tropical Medicine (ITM, Antwerp, Belgium) for further analysis.

### Data collection methods

The clinics were provided with a register dedicated for the study to record study-subject information, which included socio-demographics (name, sex, age, residence) and clinical data (case type, smear-microscopy results for three time points during treatment treatment regimen followed, and treatment outcome). HIV data could not be collected at that time, because routine counseling and testing (CT) for TB patients had not yet been implemented by 2006, and it was not logistically possible to capture this data in the study. As quality control, the study register was checked against the clinic TB registers at the end of the collection period.

### DNA extraction

To obtain genomic DNA for spoligotyping and MIRU-VNTR typing, mycobacterial colonies grown on LJ medium were resuspended in 200 μl 1 × Tris EDTA buffer (10 Mm Tris-HCl, 1 Mm Ethylenediaminetetracetic acid disodium [pH8.0]) and then boiled for 10 min. The suspension was centrifuged at 15 000 g for 1 min to pellet cell debris. The supernatant containing DNA was harvested and used in PCR reactions.

### DNA fingerprinting

Spoligotyping was performed using a commercial kit (Isogen Bioscience B.V., Maarssen, The Netherlands) according to Kamerbeek *et al. *[4]. Standardized MIRU-VNTR typing based on 15 loci was performed using the manual method [19] or by the automated method at Genoscreen in Lille, France.

### Investigation of laboratory cross contamination

The investigation of possible laboratory cross contamination or error was performed by reviewing the DNA-fingerprint patterns of clustered isolates from samples that were processed on the same day from respective laboratories.

### Fingerprint analysis

Spoligotyping and MIRU-VNTR patterns were compared to the international Spol DB4.0 database using MIRU-VNTR*plus*, a freely available web-based program [20]. This allowed assignment of shared international spoligotype numbers (ST) to known profiles. Spoligotypes that were not present in the Spol DB4.0 are referred to as 'orphan' types. MIRU-VNTR profiles with double alleles at a single locus were considered to be clonal variants of the same strain, whereas those with double alleles at 2 or more loci were considered to be mixed infections [21,22]. Identical spoligotypes and MIRU-VNTR patterns were considered to be in a cluster. Dendograms were generated using the dice coefficient and the unweighted pair group method with arithmetic averages (UPGMA). The clustering rate was defined as (*n*_c_- *c*)/*n*, where *n*_c _is the total number of clustered cases, *c *is the number of clusters, and *n *is the total number of cases in the sample. A cluster was defined as two or more patterns with identical DNA genotypes. The discriminatory power of DNA fingerprinting methods was calculated using the method described by Hunter and Gaston [23].

### Ethical Consideration

Before beginning the study, approval for the study protocol was obtained from the Ethics Committee at TDRC. In addition, approval and support was also obtained from the Director of the Ndola DHMT. The protocol was implemented in such a way as to have minimum interference with routine work at the clinics. The study did not require any additional (invasive) sampling, data collected was anonymized, there was no direct contact with the patients, and the outcome of the research data would not have an influence on patient management. As a result, we did not ask informed consent from the subjects.

### Statistical methods

Epidemiological and laboratory analysis data were double entered and descriptive analyses done in Epi Info™ (Version 3.2.2, Centers for Disease Control and Prevention, Atlanta, GA, USA). All the electronic records were manually counterchecked against the source records for completeness and consistency.

The two sided Pearson's asymptotic and exact chi square tests were appropriately used to assess associations of sex, age, geographic origin and drug-resistance profiles with spoligotyping families or MIRU-VNTR clusters using SAS^® ^9.2 (SAS Institute Inc., Cary, NC, USA.) and StatXact^® ^4.0.1 (Cytel Software Corp., Cambridge, MA, USA.). A *P *value less than 0.05 was considered statistically significant.

## Results

### Subjects and isolates

A total of 361 sputum smear-positive PTB subjects from the four selected diagnostic centres in Ndola were enrolled into the study from January to July 2006. Isolates were successfully obtained for 273 subjects, representing 54.7% (273/499) of all smear-positive PTB patients recorded in Ndola district during this period. Samples for the remaining 88 subjects included in the study, yielded either contaminated (n = 10) or negative (n = 78) cultures.

Of the cultures from 273 different subjects available for DNA fingerprinting, 85 (31.1%) were female and 188 (68.9%) were male with an age range between 14 and 79 years and a median age of 31 years. All the isolates were confirmed to be *M. tuberculosis *with an overall low drug resistance level (unpublished data).

### Characterization of *M. tuberculosis *lineages

We used spoligotyping to determine lineages of circulating *M. tuberculosis *strains in Ndola. A total of 98 different spoligotypes were obtained among the 273 isolates analyzed. Patterns from 177 isolates belonged to nine families in the Spol DB4.0, whereas 96 (35.2%) isolates could not be matched to any lineage, and are thus referred to as 'orphan'.

**Figure 1:**
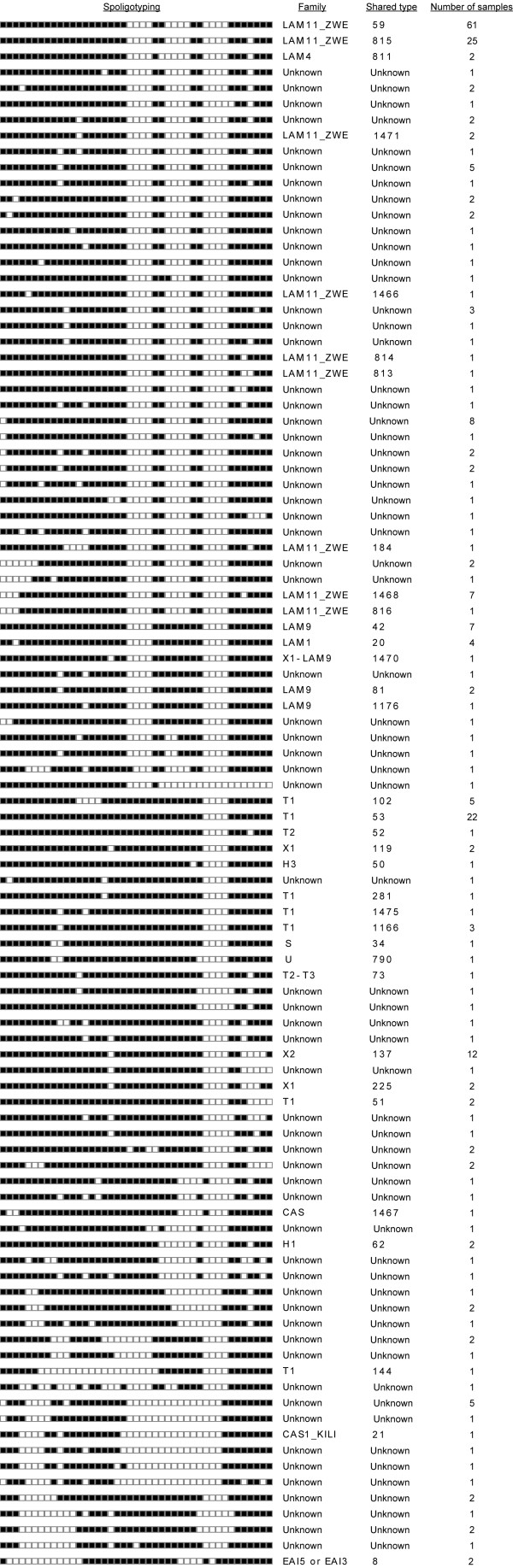
**Distinct Spoligotypes of *M. tuberculosis *isolates from Ndola**. The dendogram was generated using the dice coefficient and the unweighted pair group method with arithmetic averages (UPGMA) using the MIRU-VNTR*plus *program [20].

The largest spoligotype family was the Latin American Mediterranean (LAM) that accounted for 41.8% (114 isolates) of the total isolates, most (100 isolates) of which belonged to the LAM11_ZWE sub-family designated Southern Africa Family 1 (SAF1) [24]. The next most common family was the T family with 37 isolates (13.6%), followed by the X family at 5.9% (16 isolates). A few isolates (3.6%) belonged to the CAS, EAI, H, S, X1-LAM9 or U families (Figure 1). Although our 'orphan' isolates were not described in the Spol DB4.0, six of them showed spoligotypes identical to previously reported orphan isolates in Zambia (n = 2), Zimbabwe (n = 2) and Cape Town (n = 1) [24]. There was a uniform distribution of spoligotypes from the various study centers (data not shown). Further, no significant statistical differences were observed in the distribution with regards to age (p = 0.5073), sex (p = 0.0896) and treatment history (p = 0.1824) between the group for whom we were able to perform spoligotyping and the group for which we could not.

### Transmission analysis

To gain insight into the transmission rate of *M. tuberculosis *in Ndola, 156 (57.1%) out of the 273 samples with spoligotyping results were randomly selected and typed by 15-locus MIRU-VNTR.

**Figure 2:**
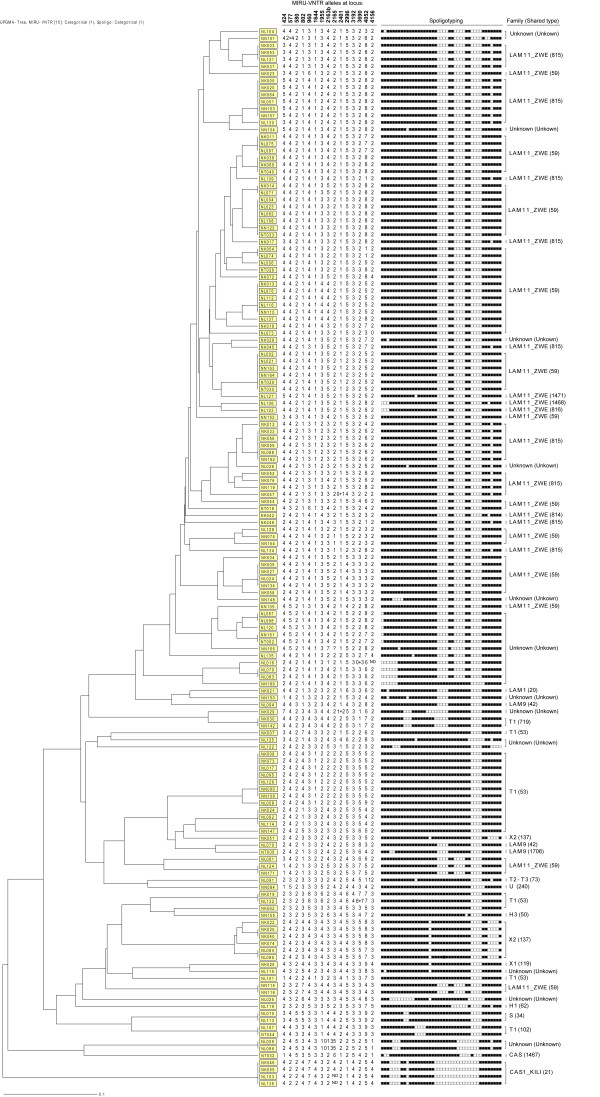
**Spoligotyping and MIRU-VNTR clustering of representative *M. tuberculosis *isolates from Ndola**. The dendogram generated using the dice coefficient and the unweighted pair group method with arithmetic averages (UPGMA) using the MIRU-VNTR*plus *program [20]. ND: Not determemined.

MIRU-VNTR analysis revealed five isolates with clonal subpopulations, i.e. the presence of double alleles at a single locus suggestive of possible ongoing evolution within a strain, and five mixed infection cases (3.2%) i.e. isolates with double alleles at 2 to 5 MIRU-VNTR loci among the 156 isolates with MIRU-VNTR results. The mixed infection cases were removed from the analysis whereas the isolates with clonal subpopulations were included in the analysis with the double alleles at a single locus treated as missing data. Thus, further analysis was performed on the 151 isolates (Figure 2).

Not surprisingly, spoligotyping alone had the lowest ability to differentiate the isolates in our sample (clustering rate of 74.2%) and a discriminatory power of 0.840. MIRU-VNTR alone yielded a clustering rate of 39.1% and a discriminatory power of 0.988. The highest discrimination was achieved when spoligotyping and MIRU-VNTR were used together (h = 0.989; clustering rate of 37.7%), which was marginally better than that of MIRU-VNTR alone (Table 1 and Figure 2).

**Table 1:** Discriminatory power of spoligotyping and 15-locus MIRU-VNTR among 151 *M. tuberculosis *isolates from Ndola, Zambia

Genotyping method	Number of different profiles	Number of isolates with unique profile	Number of clusters	Number of isolates in clusters	Clustering rate (%)	h index
Spoligotyping	39	27	12	124	74.2	0.840
MIRU-VNTR	92	65	27	86	39.1	0.988
MIRU-VNTR + spoligotyping	94	68	26	83	37.7	0.989

### MIRU-VNTR typing of SAF1 isolates

**Figure 3:**
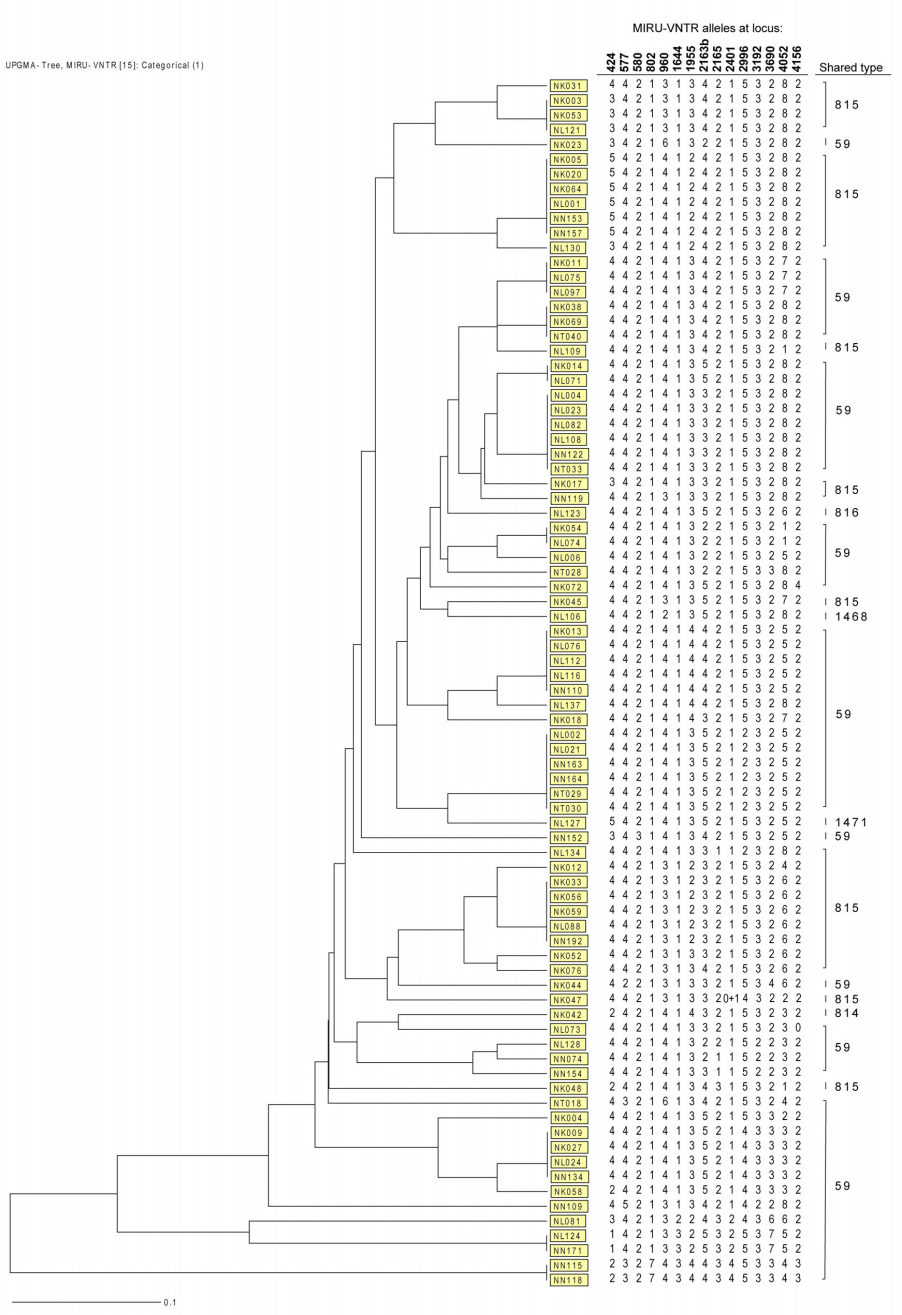
**MIRU-VNTR clustering of *M. tuberculosis *isolates belonging to the SAF1 family**. The dendogram was generated using the dice coefficient and the unweighted pair group method with arithmetic averages (UPGMA) using the MIRU-VNTR*plus *program [20].

We also assessed the genotypic similarity of isolates belonging to the major spoligotype family SAF1 among the above 151 isolates by 15-locus MIRU-VNTR. Of the 82 SAF1 isolates evaluated, MIRU-VNTR split the family into 46 different patterns i.e. 13 clusters comprising 49 isolates and 33 unique patterns (Figure 3). All isolates that were different by spoligotyping were also different by MIRU-VNTR. The differentiation of SAF1 isolates in clusters was mostly limited to one or two loci.

We did not observe any significant differences between the group of patients for whom we performed MIRU-VNTR and those we did not with regards to age (p = 0.8884), sex (p = 0.7350) and treatment history (p = 0.1536).

## Discussion

This study reports the first utilization of spoligotyping and MIRU-VNTR typing to study the diversity of *M. tuberculosis *isolates in Ndola using a large number of samples. Ndola is an urbanized city with a high prevalence of HIV (17%) and is representative of many urban towns along the line of rail in Zambia. Our results demonstrate that the SAF1 family, and to a lesser extent the T family are the main circulating TB genotypes in Ndola, causing half (50.2%) of the TB cases in the city. The predominance of ST59 and ST53 of the SAF1 has been shown to be ubiquitous in the southern African region [24,25]. The small group of genotypes accounting for most TB cases in Ndola may imply their long-standing presence in the area. The clonal variations we saw for the SAF1 family appear to support this notion.

The 15-locus MIRU-VNTR in our sample performed very well. Although the comparison with IS*6110*-RFLP was not available, the high discriminatory value achieved by MIRU-VNTR in this study suggests that the technique is suitable for studying the molecular epidemiology of TB in Ndola. The high clustering rate (37.7%) exhibited in this study suggests likely high transmission in the community that may have occurred both before and during our study period since the study included both new and retreatment cases. We did not find any evidence of laboratory cross-contamination as a possible explanation for the high cluster rate. Also, we did not observe any significant differences between age, sex and treatment history among analyzed and non-analyzed cases by either of the typing techniques. The potential association of specific genotypes or clusters with the HIV status of the patients could not be investigated because HIV data was not routinely captured by the clinics format during the time of study. Conventional epidemiological investigations of contacts could not be performed due to budgetary constraints.

Our relatively short time window of sampling (7 months) is probably still acceptable to interpret the observed clustering as resulting from 'recent transmission' [26] and might not be surprising in a high TB and HIV incidence setting. Studies on clustering rates for other African countries are rare and diverse both in methodology and results; only a few of them have used highly discriminatory typing techniques. In Botswana, 25% of investigated isolates from four communities (18 months sampling) clustered by IS*6110*-RFLP [27] whereas a population-based nationwide study with a 21 months sampling period showed a 38% cluster rate [28]. Clustering was not associated with HIV or demographic characteristics in both studies except for prior imprisonment in the first study. In Ethiopia, 41.2% of 121 isolates (12 months sampling) clustered by IS*6110 *and a clear association with HIV infection and female sex was observed [29]. In Benin, 34% of isolates (15 months sampling) clustered by 12-loci MIRU-VNTR and spoligotyping, with no parameters linked to clustering [30]. These and our cluster rates are higher than the estimated 9-13% of TB cases due to recent transmission in Malawi, where nearly half of the cases acquired TB from an HIV-positive subject [31].

Given the relatively short time window of our study compared to other African studies, our clustering rate should be considered high, and probably reflects a high recent transmission rate emphasizing the importance of early diagnosis and timely treatment. Further investigation on the link with HIV infection is required. We acknowledge that the interpretation of transmission dynamics data in this study may be limited because we did not include smear negative subjects, who are known to contribute to TB transmission [32-34].

On the one hand, these findings lend support to the premise that *M. tuberculosis *in endemic areas with predominant family strains can still possess sufficient genetic diversity when the appropriate molecular method is applied, enabling more detailed epidemiologic investigations. On the other hand, since differentiation of SAF1 isolates by 15-locus MIRU-VNTR was mostly limited to one or two loci, application of the standardized 24-locus MIRU-VNTR [35] - not used in this study due to financial constraints - may increase the ability to discriminate more MTBC among the clustered SAF1 strains.

This study also detected mixed infections in five subjects. Except for 1 of these 5 subjects, who exhibited INH resistance, isolates from the other 4 subjects, were pansusceptible to the anti-TB drugs tested. The rate of mixed infections detected in this study (at least 3.1% observed among 156 isolates with MIRU-VNTR results) is comparable to previous reports from high-incidence populations [21,22,36-38]. Although this observation does not necessarily pose a serious threat for patient management owing to the relatively low level of drug resistance in this setting, it is potentially an important factor to consider particularly for treatment of compliant subjects with unexplained changes in drug resistance patterns during the course of chemotherapy.

## Conclusion

This study has shown that the majority of MTBC isolates in Ndola belongs to the SAF1 family with a high clustering rate and that the 15-locus MIRU-VNTR typing is suitable for studying the molecular epidemiology of TB in Ndola. Finally, the probable high recent transmission rate underlines the importance of early diagnosis and timely treatment.

## Competing interests

The authors declare that they have no competing interests.

## Authors' contributions

CM was involved in the design, implementation of the study, and drafted the manuscript. ICS conceived and designed the study and critically revised the manuscript. FP critically revised the manuscript and LR was involved in the implementation and critically revised the manuscript. DK performed statistical analysis and critically revised the manuscript. NK critically revised original study design and the manuscript. All the authors read and approved the final manuscript.

## Pre-publication history

The pre-publication history for this paper can be accessed here:

http://www.biomedcentral.com/1471-2334/10/177/prepub
